# Supplementary material for: Preferences and uptake of home-based HIV self-testing for maternal retesting in Kenya
Source: PLoS One. 2024 Aug 13;19(8):e0302077. doi: 10.1371/journal.pone.0302077 (PMC11321582; doi:10.1371/journal.pone.0302077)
Supplement: S1 Table — (DOCX) [file pone.0302077.s001.docx]

|  | n (%) or median (IQR) | | | | Prevalence Ratio (95% CI) | | | |
| --- | --- | --- | --- | --- | --- | --- | --- | --- |
|  | HB-HIVST^b^(N=330) | | CB-RDT^c^ (N=664) | | Crude | p | Adjusted | p |
| Western Kenya | 330 | 168 (51) | 664 | 366 (55) | 0.89 (0.72-1.10) | 0.29 | 1.03 (0.70-1.52) | 0.89 |
| Age (year) | 330 | 23 (21-27) | 664 | 24 (21-27) | 0.99 (0.97-1.01) | 0.39 | 0.97 (0.94-1.00) | 0.08 |
| Gestational age ≥24 weeks at enrollment | 330 | 233 (71) | 664 | 491 (74) | 0.99 (0.97-1.01) | 0.39 |  |  |
| Completed secondary education | 330 | 185 (56) | 664 | 325 (49) | 1.21 (1.01-1.45) | 0.04* | 1.38 (1.04-1.85) | 0.03* |
| Employed | 330 | 104 (32) | 664 | 231 (35) | 0.91 (0.75-1.10) | 0.31 |  |  |
| Household income ≥10,000 (KSH) per month | 295 | 125 (42) | 581 | 193 (33) | 1.29 (1.06-1.58) | 0.01* | 1.43 (1.02-2.01) | 0.04* |
| Depression^a^ | 330 | 166 (50) | 664 | 381 (57) | 0.83 (0.69-1.00) | 0.05* | 0.79 (0.56-1.12) | 0.18 |
| Have live births | 330 | 172 (52) | 664 | 375 (56) | 0.89 (0.75-1.06) | 0.19 |  |  |
| Current pregnancy intended | 328 | 196 (60) | 663 | 386 (58) | 1.04 (0.85-1.27) | 0.68 |  |  |
| Relationship | 330 |  | 664 |  |  |  |  |  |
| Married^b^ |  | 284 (86) |  | 548 (83) | ref | ref |  |  |
| No partner |  | 21 (6) |  | 46 (7) | 0.92 (0.64-1.32) | 0.65 |  |  |
| Not married w partner |  | 25 (8) |  | 70 (11) | 0.77 (0.54-1.09) | 0.14 |  |  |
| Low partnership power^c^ | 297 | 79 (27) | 598 | 147 (25) | 1.07 (0.87-1.33) | 0.52 |  |  |
| Ever tested with STI | 326 | 5 (2) | 662 | 22 (3) | 0.55 (0.25-1.24) | 0.15 |  |  |
| Polygamous^+^ | 309 | 8 (3) | 616 | 46 (7) | 0.43 (0.22-0.82) | 0.01* |  |  |
| Traveling time to clinic ≥1 hour^i^ | 330 | 83 (25) | 663 | 125 (19) | 1.27 (1.03-1.57) | 0.03* | 1.39 (0.94-2.06) | 0.1 |
| Using transportation to clinic^d^ | 329 | 223 (68) | 663 | 473 (71) | 0.89 (0.74-1.09) | 0.26 |  |  |
| Waiting time ≥1 hour at clinic^d^ | 330 | 128 (39) | 663 | 206 (31) | 1.25 (1.05-1.49) | 0.01* | 1.27 (0.91-1.79) | 0.16 |
| Ever left clinic because of long wait | 329 | 46 (14) | 664 | 79 (12) | 1.13 (0.88-1.44) | 0.33 |  |  |
| Schedule not working with clinic hours | 330 | 56 (17) | 664 | 67 (10) | 1.45 (1.16-1.81) | <0.01* | 2.26 (1.44-3.57) | <0.01* |

Interquartile range (IQR); home-based self-testing (HB-HIVST); clinic-based testing (CB-RDT); prevalence ratio (PR); confidence interval (CI). a. score of >10 on Edinburgh Postnatal Depression Scale (EDPS); b. married / cohabitating (vs. no partner); c. score in lowest quantile (<2.15) on Sexual Relationship Power Scale (SRPS); d. at last clinic visit before enrollment

* p<0.05; ** Includes as interaction term with preterm birth. Kenya Shilling (KSH) ~ $1 USD.

+. Polygamy was excluded in the adjusted model due to missing data
