# Supplementary material for: Preferences and uptake of home-based HIV self-testing for maternal retesting in Kenya
Source: PLoS One. 2024 Aug 13;19(8):e0302077. doi: 10.1371/journal.pone.0302077 (PMC11321582; doi:10.1371/journal.pone.0302077)
Supplement: S2 Table — (DOCX) [file pone.0302077.s002.docx]

|  | Retested with HB-HIVST (N=121) | | Retested with CB-RDT (N=174) | | Crude PR (95% CI) | p |
| --- | --- | --- | --- | --- | --- | --- |
|  | N, median (IQR) or n (%) | | | |  |  |
| Western Kenya | 121 | 47 (39) | 174 | 91 (52) | 0.71 (0.53-0.95) | 0.02* |
| Age (years) | 121 | 24 (22-27) | 174 | 23 (21-27) | 1.01 (0.99-1.04) | 0.31 |
| Gestational age ≥24 weeks at enrollment | 121 | 86 (72) | 174 | 129 (74) | 0.94 (0.69-1.28) | 0.70 |
| Preterm birth (<37 weeks gestation age at delivery)  Tested in pregnancy  Tested in postpartum | 120 | 27 (22) | 174 | 70 (40) | 0.83 (0.35-1.94)  0.29 (0.15-0.57) | 0.67  <0.001* |
| Tested during postpartum (ref: pregnancy/delivery) | 120 | 84 (70) | 174 | 99 (57) | ** | ** |
| Completed secondary education | 121 | 67 (55) | 174 | 102 (59) | 0.91 (0.69-1.20) | 0.51 |
| Employed | 121 | 41 (34) | 174 | 56 (32) | 1.06 (0.79-1.42) | 0.70 |
| Household income ≥10,000 (KSH) per month | 107 | 56 (52) | 158 | 62 (39) | 1.40 (1.04-1.87) | 0.03* |
| Depression^a^ | 121 | 47 (39) | 174 | 97 (56) | 0.64 (0.48-0.86) | <0.001* |
| Have live births | 121 | 73 (60) | 174 | 88 (51) | 1.25 (0.94-1.66) | 0.13 |
| Current pregnancy intended | 121 | 74 (61) | 172 | 103 (60) | 1.02 (0.78-1.32) | 0.89 |
| Married/cohabitating^b^ | 121 | 109 (91) | 174 | 148 (85) | 1.47 (0.88-2.44) | 0.14 |
| Relationship duration <1 year^b^ | 116 | 10 (9) | 162 | 22 (14) | 0.73 (0.44-1.21) | 0.22 |
| Low partnership power^c^ | 117 | 31 (26) | 161 | 43 (27) | 1.02 (0.74-1.40) | 0.92 |
| Traveling time to clinic ≥1 hour^d^ | 121 | 27 (22) | 174 | 49 (28) | 0.84 (0.6-1.16) | 0.29 |
| Using transportation to clinic^d^ | 120 | 75 (62) | 174 | 122 (70) | 0.81 (0.61-1.07) | 0.14 |
| Waiting time ≥1 hour at clinic^d^ | 121 | 51 (42) | 174 | 64 (37) | 1.16 (0.88-1.53) | 0.30 |
| Ever left clinic because of long wait | 121 | 21 (17) | 174 | 20 (12) | 1.31 (0.92-1.87) | 0.14 |
| Schedule not working with clinic hours | 121 | 19 (16) | 174 | 27 (16) | 1.02 (0.69-1.49) | 0.93 |
| Partner tested for HIV during follow-up^e^ | 115 | 84 (73) | 155 | 82 (53) | 1.70 (1.22-2.36) | <0.001* |

Home-based self-testing (HB-HIVST); clinic-based testing (CB-RDT); Interquartile range (IQR); prevalence ratio (PR); confidence interval (CI); a. assessed by Edinburgh Postnatal Depression Scale (EDPS) with a score of >10; b. married / cohabitating (vs. no partner); c. score in lowest tertile (<2.15) on Sexual Relationship Power Scale (SRPS); d. assessed with the last clinic visit before enrollment; e. among women who had an HIV-negative or unknown partner and reported partner testing status during follow-up. Kenya Shilling (KSH) ~ $1 USD.

* p<0.05; ** Includes as interaction term with preterm birth
