# Supplementary material for: Preferences and uptake of home-based HIV self-testing for maternal retesting in Kenya
Source: PLoS One. 2024 Aug 13;19(8):e0302077. doi: 10.1371/journal.pone.0302077 (PMC11321582; doi:10.1371/journal.pone.0302077)
Supplement: S3 Table — (DOCX) [file pone.0302077.s003.docx]

|  | Retested with HB-HIVST (N=108) | | Retested with CB-RDT (N=143) | | Crude PR (95% CI) | p |
| --- | --- | --- | --- | --- | --- | --- |
|  | N, median (IQR) or n (%) | | | |  |  |
| Western Kenya | 108 | 44 (41) | 143 | 69 (48) | 0.84 (0.63 - 1.12) | 0.24 |
| Age (years) | 108 | 24 (22, 28) | 143 | 23 (21, 27) | 1.02 (0.99 - 1.04) | 0.19 |
| Gestational age ≥28 weeks at enrollment | 108 | 54 (50) | 143 | 67 (47) | 1.07 (0.8 - 1.43) | 0.63 |
| Preterm birth (<37 weeks gestation age at delivery)  Tested in pregnancy  Tested in postpartum | 108 | 22 (20) | 142 | 56 (39) | 0.85 (0.32-2.27)  0.26 (0.12-0.53) | 0.74  <0.001* |
| Tested during postpartum (ref: pregnancy/delivery) | 108 | 77 (71%) | 142 | 87 (61%) | ** | ** |
| Completed secondary education | 108 | 57 (53) | 143 | 86 (60) | 0.84 (0.64 - 1.12) | 0.24 |
| Employed | 108 | 40 (37) | 143 | 44 (31) | 1.17 (0.88 - 1.56) | 0.29 |
| Household income ≥10,000 (KSH) per month | 95 | 49 (52) | 131 | 52 (40) | 1.32 (0.97 - 1.78) | 0.07 |
| Depression^a^ | 108 | 43 (40) | 143 | 79 (55) | 0.7 (0.52 - 0.94) | 0.02 |
| Have live births | 108 | 66 (61) | 143 | 75 (52) | 1.23 (0.91 - 1.65) | 0.18 |
| Current pregnancy intended | 108 | 65 (60) | 142 | 86 (61) | 0.99 (0.76 - 1.3) | 0.95 |
| Married/cohabitating^b^ | 108 | 98 (91) | 143 | 123 (86) | 1.33 (0.8 - 2.21) | 0.27 |
| Relationship duration <1 year^b^ | 104 | 8 (8) | 132 | 18 (14) | 0.67 (0.38 - 1.19) | 0.18 |
| Low partnership power^c^ | 104 | 25 (24) | 132 | 34 (26) | 0.95 (0.67 - 1.34) | 0.77 |
| Traveling time to clinic ≥1 hour^d^ | 108 | 21 (19) | 143 | 43 (30) | 0.71 (0.49 - 1.02) | 0.07 |
| Using transportation to clinic^d^ | 107 | 69 (64) | 143 | 98 (69) | 0.9 (0.68 - 1.19) | 0.47 |
| Waiting time ≥1 hour at clinic^d^ | 108 | 46 (43) | 143 | 57 (40) | 1.07 (0.8 - 1.42) | 0.66 |
| Ever left clinic because of long wait | 108 | 20 (19) | 143 | 17 (12) | 1.31 (0.92 - 1.87) | 0.13 |
| Schedule not working with clinic hours | 108 | 17 (16) | 143 | 21 (15) | 1.05 (0.7 - 1.56) | 0.82 |
| Partner tested for HIV during follow-up^e^ | 103 | 73 (71) | 129 | 69 (53) | 1.54 (1.11 - 2.15) | 0.01* |

Home-based self-testing (HB-HIVST); clinic-based testing (CB-RDT); Interquartile range (IQR); prevalence ratio (PR); confidence interval (CI); a. assessed by Edinburgh Postnatal Depression Scale (EDPS) with a score of >10; b. married / cohabitating (vs. no partner); c. score in lowest tertile (<2.15) on Sexual Relationship Power Scale (SRPS); d. assessed with the last clinic visit before enrollment; e. among women who had an HIV-negative or unknown partner and reported partner testing status during follow-up. Kenya Shilling (KSH) ~ $1 USD.

* p<0.05; ** Includes as interaction term with preterm birth
