# Supplementary material for: Preferences and uptake of home-based HIV self-testing for maternal retesting in Kenya
Source: PLoS One. 2024 Aug 13;19(8):e0302077. doi: 10.1371/journal.pone.0302077 (PMC11321582; doi:10.1371/journal.pone.0302077)
Supplement: S4 Table — (DOCX) [file pone.0302077.s004.docx]

|  | Crude PR (95% CI) | p-value | Adjusted PR (95% CI) | p-value |
| --- | --- | --- | --- | --- |
| Western Kenya site (vs Nairobi site) | 0.70 (0.49-0.99) | 0.04* | 1.13 (0.64-2.02) | 0.67 |
| Age (years) | 1.02 (0.99-1.06) | 0.15 | 1.02 (0.97-1.07) | 0.51 |
| Gestational age ≥24 weeks at enrollment | 0.90 (0.64-1.27) | 0.56 |  |  |
| Secondary education completed | 1.12 (0.80-1.57) | 0.50 |  |  |
| Employed | 1.21 (0.86-1.69) | 0.27 |  |  |
| Household income ≥10,000 (KSH) per month | 1.72 (1.21-2.44) | <0.01* | 1.57 (0.99-2.49) | 0.05 |
| Tested during postpartum (ref: pregnancy) | 1.16 (0.81-1.66) | 0.41 | ** | ** |
| Preterm birth (<37 weeks gestation age at delivery)  Tested in pregnancy  Tested in postpartum | 0.58 (0.38-0.87) | 0.01* | 0.96 (0.37-2.49)  0.32 (0.17-0.60) | 0.94  <0.001* |
| Depression^a^ | 0.51 (0.36-0.72) | <0.01* | 0.40 (0.24-0.67) | <0.001* |
| Have live births | 1.09 (0.78-1.52) | 0.61 |  |  |
| Current pregnancy intended | 0.99 (0.70-1.38) | 0.93 |  |  |
| Married/cohabitating^b^ | 1.68 (0.96-2.95) | 0.07 | 1.53 (0.58-4.03) | 0.39 |
| Relationship duration <1 year | 0.63 (0.33-1.21) | 0.17 |  |  |
| Low partnership power^c^ | 1.06 (0.72-1.56) | 0.77 |  |  |
| Ever diagnosed with STI | 0.36 (0.05-2.42) | 0.29 |  |  |
| Traveling time to clinic ≥1 hour^d^ | 0.83 (0.54-1.28) | 0.40 |  |  |
| Used transportation to travel to clinic^d^ | 0.75 (0.53-1.06) | 0.11 |  |  |
| Waiting time ≥1 at clinic^d^ | 1.37 (0.99-1.92) | 0.06 | 1.31 (0.84-2.05) | 0.23 |
| Ever left clinic because of long wait | 1.48 (0.97-2.25) | 0.07 | 1.56 (0.84-2.87) | 0.16 |
| Schedule not working with clinic hours | 1.45 (0.92-2.28) | 0.11 |  |  |
| Partner tested for HIV during follow-up^e^ | 2.96 (2.03-4.31) | <0.01* | 4.67 (2.71-8.04) | <0.001* |

Prevalence ratio (PR); confidence interval (CI); a. assessed by Edinburgh Postnatal Depression Scale (EDPS) with a score of >10; b. married / cohabitating (vs. no partner); c. score in lowest quantile (<2.15) on Sexual Relationship Power Scale (SRPS); d. assessed with the last clinic visit before enrollment; e. among women who had an HIV-negative or unknown partner and reported partner testing status during follow-up. Kenya Shilling (KSH) ~ $1 USD.

* p<0.05; ** Included as interaction term with preterm birth
