# Supplementary figures and images for: Preferences and uptake of home-based HIV self-testing for maternal retesting in Kenya
Source: PLoS One. 2024 Aug 13;19(8):e0302077. doi: 10.1371/journal.pone.0302077 (PMC11321582; doi:10.1371/journal.pone.0302077)

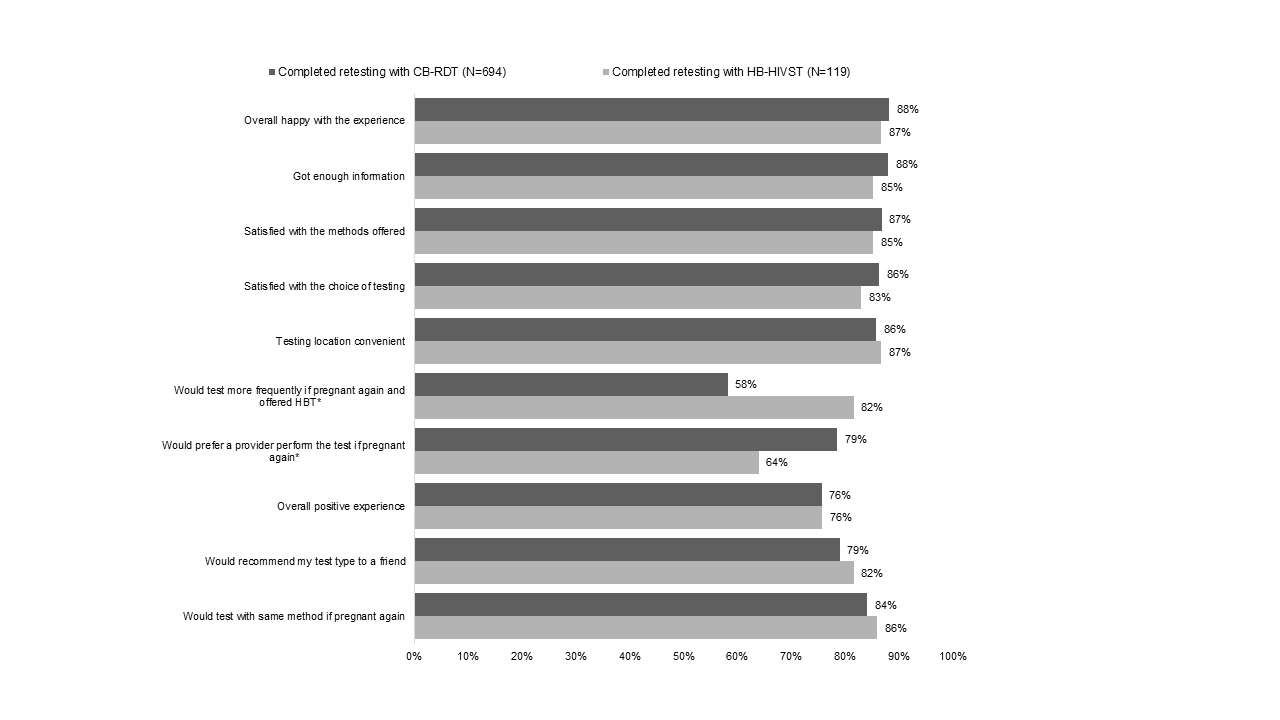

Supplement: S1 Fig — Questions were answered based on self-reported responses of “Strongly Agree” or “Agree”. * p<0.05 by Chi-square test. Home-based HIV self-test (HB-HIVST); clinic-based rapid diagnostic test (CB-RDT). (TIF) [file pone.0302077.s005.tif]
