# Supplementary material for: Preferences and uptake of home-based HIV self-testing for maternal retesting in Kenya
Source: PLoS One. 2024 Aug 13;19(8):e0302077. doi: 10.1371/journal.pone.0302077 (PMC11321582; doi:10.1371/journal.pone.0302077)
Supplement: Attachment — Submitted filename: Reviewers comments-PONE_BN.1.5.24.pdf [file pone.0302077.s006.pdf]

Reviewer: Bernard Njau

Title "**Preferences and Uptake of Home-based HIV Self-testing for Maternal Retesting in Kenya**".

Manuscript ID: PONE-D-24-11702

General Comment:

This research is relevant in the field of HIV/AIDS care and treatment among pregnant women attending antenatal clinics in Kenya. The study is well thought out, designed, and implemented; however, the authors need to address the following comments for improvement:

Abstract:

Results;

i). The authors should present the sample(n=) before presenting the percentages (%) or vice versa. This should be done in the result section as well.

Conclusion:

i) The authors should be more specific in their conclusions based on their study objectives and key findings, and provide specific recommendations for their conclusions. I suggest the authors to recast their conclusion.

Main Text:

Background.

i) In the last paragraph of the background section the authors should add what are the expected benefits of the study findings for pregnant women, their partners, and other secondary beneficiaries.

Methods

i) The authors have used MCH…is it not Reproductive and Child Health(RCH)clinics? See line 24 on page 4.

Ethical consideration;

ii) Authors should provide ethical clearance certificate identification numbers and date/month/year. See lines 2 to 4 on page 5.

Data collection;

i) The authors should describe the source of the questionnaire use and the number of questions (n=? open-ended; n=? close-ended?).

ii) Number of study nurses? Were they trained? How long? On what? What was their previous experience in such research?

Measurement;

i) Authors should add the measurement sub-section and provide sample questions and expected responses for all categories of independent variables and dependent variables.

ii) Measurement for internal variability?

iii) Was piloting testing of the data collection tools done? Among how many samples? What were the aims of doing the piloting and how were the results utilized?

iv) The authors mentioned "saliva"…I'm not sure if it's correct to mention saliva as the sample used in Oral HIVST…I suggest authors to use the correct word. See line 4 on page 6.

v) The authors mentioned" …. with test conduct and interpretation…" What method/strategy did the authors use to ascertain this observation? See lines 6 & 7 on page 6.

vi) The authors mentioned, "Participants were asked to 'flash'…". What about those who did not flash? For how long (hours? days? Weeks?) did the nurses wait for the flash before making a follow-up? How frequently did the nurses attempt to make follow-ups before stopping? See lines 22-23 and lines 6 -9 on page 7.

vii) In the last paragraph under statistical analysis, I suggest the authors add a sentence "We used adjusted Prevalence ratio with their corresponding 95% Confidence Interval to summarize the strength of the association between the independent variables and the dependent variable(s).

Results:

i) Authors should refer to my early comment in the abstract section.

ii) Authors should give the reason for not enrolling the 3 eligible participants.

iii) What was the attrition rate?

iv) Authors should present their findings under the following sub-sections, a) Descriptive findings, b) Bivariate analysis findings, and c) multivariable analysis findings for a reader to follow.

v) The authors mentioned: "…completing secondary education…, and inconvenient clinic hours…..." However, these two observation were not reported in the abstract section. See lines 13 -15 on page 9.

Discussion:

i) The authors mentioned that" HB-HIVST was highly acceptable…" What was the reference of acceptability to justify this statement? See line 19 on page 11.

ii) The following sentence" These findings demonstrate a current preference and better uptake for CB-RDT, but also barriers to HB-HIVST" not convincing. To my understanding, HIVST helps to circumvent barriers associated with facilities-based HTC. I suggest the authors should provide a plausible explanation for the CB-RDT preference in this study setting. See the lines 23-24 on page 11.

iii) The authors mentioned,"…other logistical or behavioral factors…". I suggest the authors provide examples of those factors. See line 2 on page 13.

Study limitations:

i). The authors should report methodological study limitations, and steps used to minimize the limitations (e.g., self-reporting of HIVST results? Attrition rate? study design? etc.).
